# Une complication musculaire rarissime de l'hémophilie: la myosite ossifiante

**DOI:** 10.11604/pamj.2015.22.149.7909

**Published:** 2015-10-16

**Authors:** Samia Frioui, Sonia Jemni

**Affiliations:** 1 Service de Médecine Physique et de Réadaptation Fonctionnelle, CHU Sahloul Sousse, Tunisie

**Keywords:** Myosite ossifiante, hémophilie A, os, muscle, TDM, myositis ossificans, hemophilia A, bone, muscle, CT scan

## Image en médecine

La myosite ossifiante circonscrite est une affection bénigne caractérisée par une prolifération hétérotopique d'os dans les tissus mous. Elle survient généralement chez des adolescents ou de jeunes adultes. De très rares cas ont été décrits chez les enfants. Son étiopathogénie n'est pas claire, il semble toutefois qu'un foyer de nécrose musculaire ou un hématome puissent être à l'origine de la lésion. Les sièges les plus fréquents sont le quadriceps et le biceps brachial. Nous présentons le cas d'un patient âgé de vingt ans, aux antécédents d'hémophilie A, admis dans notre service de Médecine Physique et de Réadaptation Fonctionnelle pour prise en charge d'une limitation de la mobilité des deux hanches. L'histoire remonte à un an où le patient a présenté suite à une chute de sa propre hauteur avec réception sur les hanches une douleur et une tuméfaction à leur niveau. L’évolution était marquée par l'installation d'une limitation de la mobilité des deux hanches responsable d'une gêne fonctionnelle et d'une réduction du périmètre de marche. L'examen clinique objectivait une limitation douloureuse de la mobilité des hanches sans autre signe accompagnateur. Les radiographies standards (A, B,C) et le scanner du bassin (D, E) ont montré une ossification musculaire autour des deux hanches, aspect compatible avec une myosite ossifiante progressive des muscles carrés fémoraux des deux côtés et du jumeau inférieur droit. Une intervention chirurgicale a été proposée mais refusée par le patient. Il a alors bénéficié d'un traitement médical et de rééducation à visée antalgique. L’évolution était marquée par une légère amélioration fonctionnelle.

**Figure 1:**
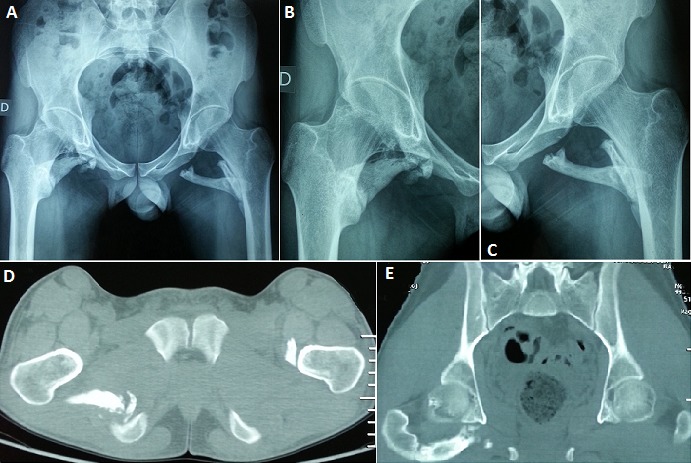
A) radiographie du bassin de face: calcifications se projetant au niveau des parties molles des deux hanches sans lyse osseuse en regard; B) radiographie de la hanche droite de face: calcifications se projetant au niveau des parties molles sans lyse osseuse en regard; C) radiographie de la hanche gauche de face: calcifications se projetant au niveau des parties molles sans lyse osseuse; D) coupe axiale scannographique au niveau des articulations coxo-fémorales: ossification musculaire autour des deux hanches, compatible avec une myosite ossifiante des muscles carrés fémoraux des deux côtés et du jumeau inférieur droit; E) coupe axiale scannographique au niveau des articulations coxo-fémorales: ossification musculaire autour des deux hanches, compatible avec une myosite ossifiante des muscles carrés fémoraux des deux côtés et du jumeau inférieur droit

